# Bridging the data gaps: do we have the right balance between country data and global estimates?

**DOI:** 10.1080/16549716.2017.1299978

**Published:** 2017-05-22

**Authors:** Carla AbouZahr, Ties Boerma, Peter Byass

**Affiliations:** ^a^ CAZ Consulting, Geneva, Switzerland; ^b^ Department of Information, Evidence and Research, WHO, Geneva, Switzerland; ^c^ Department of Community Health Sciences, University of Manitoba, Winnipeg, Canada; ^d^ Department of Public Health and Clinical Medicine, Umeå University, Umeå, Sweden; ^e^ School of Public Health, University of the Witwatersrand, Johannesburg, South Africa; ^f^ Institute of Applied Health Sciences, University of Aberdeen, Aberdeen, UK

We fondly remember Hans Rosling and his informative and entertaining bubble charts [1]. The presentation of statistics in so congenial a manner can nevertheless divert attention from the sources of all those health and development indicators, for so many countries, and spanning such long time periods. The reality is that the rich statistical series encapsulated in those comparative charts were based on mathematical estimates derived from such data as were available, mostly developed by international agencies and academics based in high-income countries [2].

Even though health data availability has improved dramatically during recent decades, especially through household surveys, there are still major gaps in the supply of high-quality and timely information. The ever-growing demand for up-to-date comparable health statistics is not matched by an increasing supply of reliable data on population health indicators, such as mortality and causes of death [3].

To compensate for such limitations, analysts integrate available data into statistical models, which borrow heavily from the historical basis of data in rich countries, in order to generate plausible time trends and predict current indicator values for all countries. These models incorporate whatever country data are available and produce estimates by making assumptions about the evolution of indicators over time and in response to changing conditions such as household wealth, education, life expectancy, environmental change etc. Hans Rosling’s *GapMinder* (www.gapminder.org) relies on the resulting estimates to disseminate convincing analyses of the past and persuasive visions of future health, thus informing and influencing the international and national discourse on health priorities.

Specialised United Nations (UN) agencies such as the United Nations Population Fund (UNFPA), the World Health Organization (WHO) and the United Nations Children’s Fund (UNICEF) have been producing estimates of population and health indicators for many decades, often working with academia. In 2008, the Seattle-based Institute for Health Metrics and Evaluation (IHME) entered the field, and has the unwavering support of the Bill & Melinda Gates Foundation, which recently announced a commitment to invest a further $279 million in IHME’s estimation work over the next decade [4]. Such investments enable the production of any health statistic for any country at any point in time. Estimates based on modelled predictions primarily meet the demand of global development partners and donor agencies for up-to-date comparable statistics for today, and sometimes the future.

But for countries and at sub-national levels, those responsible for taking policy decisions are not always as enamoured of the estimates as is the global health community. The preference is for working with local personnel, using country-based data sets derived from national surveys and local administrative systems, such as the health facility information system or administrative systems such as the registration of births and deaths. The data may well be imperfect – incomplete, inaccurate, or out-of-date. Nonetheless, results can be locally and socially relevant and data producers can readily describe to decision-makers how they are generated and explain their limitations. By contrast, estimates produced by global agencies emerge from a ‘black box’ that is hard to understand and describe to non-experts, giving them limited value from a policy-maker’s perspective. Efforts to explain and interpret statistical uncertainty intervals around global estimates often fall on deaf ears, and are of little value in in-country discussions [5]. Adoption of the Guidelines for Accurate and Transparent Health Estimates Reporting (GATHER) for transparent reporting of estimates through better sharing of data, model specifications, assumptions and computer code may help in this respect, at least among technical experts [6].

Over the past few years there have been many calls for increased investments in enhancing country health measurement and monitoring systems and capacities. Responses have often been muted and short-lived. It is rare to find ongoing global support for development of country information systems such as sample registration and civil registration and vital statistics. For instance, Tanzania had to close down its highly effective and low-cost national sample registration system because the main donor’s interest moved into other areas of data collection. Support to the well-established INDEPTH Network of health and demographic surveillance sites is wavering and sometimes replaced by parallel investments [7]. Expensive single-topic household surveys are still common, driven by donor needs, rather than country demand for comprehensive epidemiological data that capture all leading health challenges. Ambitious international and country efforts to improve health facility information systems are always at risk of underfunding and fragmentation as they are driven by immediate project needs – and sometimes even by donors’ desires for short-term kudos. And finally, domestic investments have been piecemeal. A rare example of long-term strong support is the USAID-supported Demographic & Health Survey (DHS) programme, which has become the backbone of population health data for dozens of low and lower-middle income countries for almost three decades [8]. Notwithstanding the value of the DHS programme, repeated cross-sectional household surveys with different random samples cannot alone meet all country needs, especially for local-level and continuous data.

There is little disagreement about the diagnosis of the problem of fragmentation and inefficiencies. In 2016 the Health Data Collaborative (www.healthdatacollaborative.org) was established to try to address this issue, with a broad agenda of strengthening country health information systems. This will be a perilous journey: large well-funded disease-specific programmes and projects can afford to invest in generating the data they need for their own monitoring and evaluation, but rarely contribute to broad-based systems building.

This Special Issue of *Global Health Action* presents a range of papers that speak to various aspects of the intrinsic challenges facing country-based health information systems. An introductory paper considers the usefulness of the expanding volume of global estimates [9]. Three country-specific papers consider the role of estimates in the specific contexts of Chile, Bangladesh and Thailand, respectively [10–12]. Pisani and Kok reflect on the need to socially contextualise estimates in order to make them locally useful [13]. Mahy and colleagues describe the process of generating global HIV estimates, which is often cited as the gold standard for other public health estimates in terms of country involvement, ownership and capacity building [14]. Finally, the transparency of global estimates made before the adoption of the GATHER principles is reviewed [15].

So where should we go from here? Are we destined to maintain two parallel but unequal pathways: donors and development agencies focused on the indisputable value of globally produced estimates, while country players struggle to build sustainable, country-led health information and statistical systems? The massive investments in estimation and advances in presentation of results, such as pretty geospatial maps, may be useful for better targeting of public health interventions but also run the risk of amplifying the dichotomy.

Very few non-specialists understand the differences between a modelled prediction or estimate and empirical statistics. It surely cannot be right that countries appear to serve as mere sources of the raw materials that go into global statistical models. What is needed is a distribution of resources that would enable countries to become equal partners in global efforts to improve our understanding of health and development at global, national and local levels. Had such investments been forthcoming throughout the Millennium Development Goals (MDG) era, countries might have been better placed to tackle the more complex current statistical demands of the Sustainable Development Goals (SDG) [16].
